# The association between glycated hemoglobin, physical activity and infertility: a multiple logistic regression and mediation analysis based on the NHANES database

**DOI:** 10.3389/fendo.2025.1495470

**Published:** 2025-03-26

**Authors:** Panpan Chen, Leilei Zhu, Yanli Mou, Shuyun Zhao, Guanyou Huang

**Affiliations:** ^1^ Reproductive Medicine Center, Department of Obstetrics and Gynecology, Affiliated Hospital of Guizhou Medical University, Guiyang, China; ^2^ Reproductive Medicine Center, Affiliated Jinyang Hospital of Guizhou Medical University, Guiyang, China

**Keywords:** physical activity (PA), activity patterns, infertility, glycated hemoglobin (HbA1c), mediation

## Abstract

**Objective:**

To determine the relationship between physical activity (PA) patterns and infertility among women in the United States and to ascertain whether glycated hemoglobin (HbA1c) influences this association.

**Design:**

A cross-sectional study of PA patterns and infertility was conducted. The PA classification was based on two categories: recreational PA and work-related PA. The subgroups were classified as inactive, less active, or active. We conducted data analysis via both multiple logistic regression analysis and mediation analysis.

**Setting:**

National Health and Nutrition Examination Survey (NHANES, 2013–2018).

**Participants:**

Women aged 18–49 years (n, 3,948).

**Results:**

There was no statistically significant link demonstrated between infertility and work-related PA patterns. The recreational PA group may decrease the prevalence of infertility, with its occurrence being merely 0.79 times that of inactive group. (95% CI: 0.62, 1.0; *p* = 0.045). The consistency of this discrepancy was not preserved in Model III after the mixed effect was incorporated into the recreational PA group. The stratified research findings revealed that the association between recreational PA patterns and infertility was influenced by variables such as age, BMI, and history of diabetes. Furthermore, the mediation analysis revealed that recreational PA did not have a statistically significant direct effect on infertility (*p* = 0.098). HbA1c serves as a mediator in this interaction (95% CI: -0.06, -0.02).

**Conclusion:**

Recreational PA patterns were associated with infertility among women aged 18–49 years in the United States, which was likely due to the effects of HbA1c.

## Introduction

Infertility is characterized as the inability to conceive following 12 months of consistent, unprotected sexual intercourse (1). This condition is a major worldwide health concern that adversely affects individual well-being and human rights. The most recent guidelines from the World Health Organization (WHO) indicate that approximately 10–15% of couples in the United States experience infertility, with the frequency of infertility increasing with age (2). Infertility is influenced by a variety of complex circumstances involving both male and female contributions. WHO guidelines identify the primary contributors to female infertility as ovulatory disorders, pelvic factors, infectious factors, immunological factors, and unexplained causes. In females, age is a paramount factor influencing fertility, as the ovarian reserve decreases with advancing age, thus resulting in a reduction in both egg quality and quantity (3). Moreover, the incidence of aneuploidy (which stems from chromosomal irregularities) is more prevalent in older women, thus increasing the likelihood of miscarriage and contributing to infertility (4). This underscores the necessity for an extensive understanding of female reproductive health, especially regarding the impact of various lifestyle factors on fertility.

PA is essential for sustaining overall health. The WHO defines PA as any bodily action that expends energy through the skeletal muscular system. Regular PA has numerous advantages, such as improved cardiovascular function, effective weight management, enhanced metabolic health, and increased mental well-being (5). These factors may indirectly affect fertility by regulating hormone balance and enhancing reproductive outcomes (6, 7).

HbA1c is an essential biomarker for evaluating long-term glucose regulation, especially in individuals with associations with diabetes. Recent studies have indicated that HbA1c may also represent overall metabolic health and can act as a marker for reproductive health (8, 9). Recent studies have also shown that an increase in HbA1c levels is significantly associated with the risk of ovarian dysfunction, which may affect ovarian health by influencing insulin resistance and the inflammatory response (10). Regular PA has been shown to lower HbA1c levels, thereby improving metabolic health and improving ovarian function (11). This effect is particularly pertinent for women, due to the fact that increased HbA1c levels have been linked to unfavorable reproductive outcomes, including heightened risks of conception challenges and hazardous pregnancy problems such as gestational diabetes mellitus, early pregnancy abortion, fetal dysplasia, etc (12). Additional data have indicated correlations among metabolic health, physical fitness, and reproductive health (13). Nevertheless, there are limited findings regarding the mediation function of HbA1c in the relationship between PA and infertility. Therefore, an understanding of the relationships among PA, HbA1c levels, and infertility may yield significant insights into prospective therapeutic options for enhancing reproductive health in aging women. This article categorized PA levels into three groups, including active, less active, and inactive groups, according to PA guidelines (14–16). A stratified study was performed to examine the associations between infertility and PA levels, as well as explore the mediating role of HbA1c in the relationship between PA and infertility, with a particular focus on female reproductive health. This analysis aims to understand how lifestyle modifications can enhance fertility outcomes and inform clinical practices for those individuals who experience infertility.

## Materials and methods

### Study population

**Figure 1:**
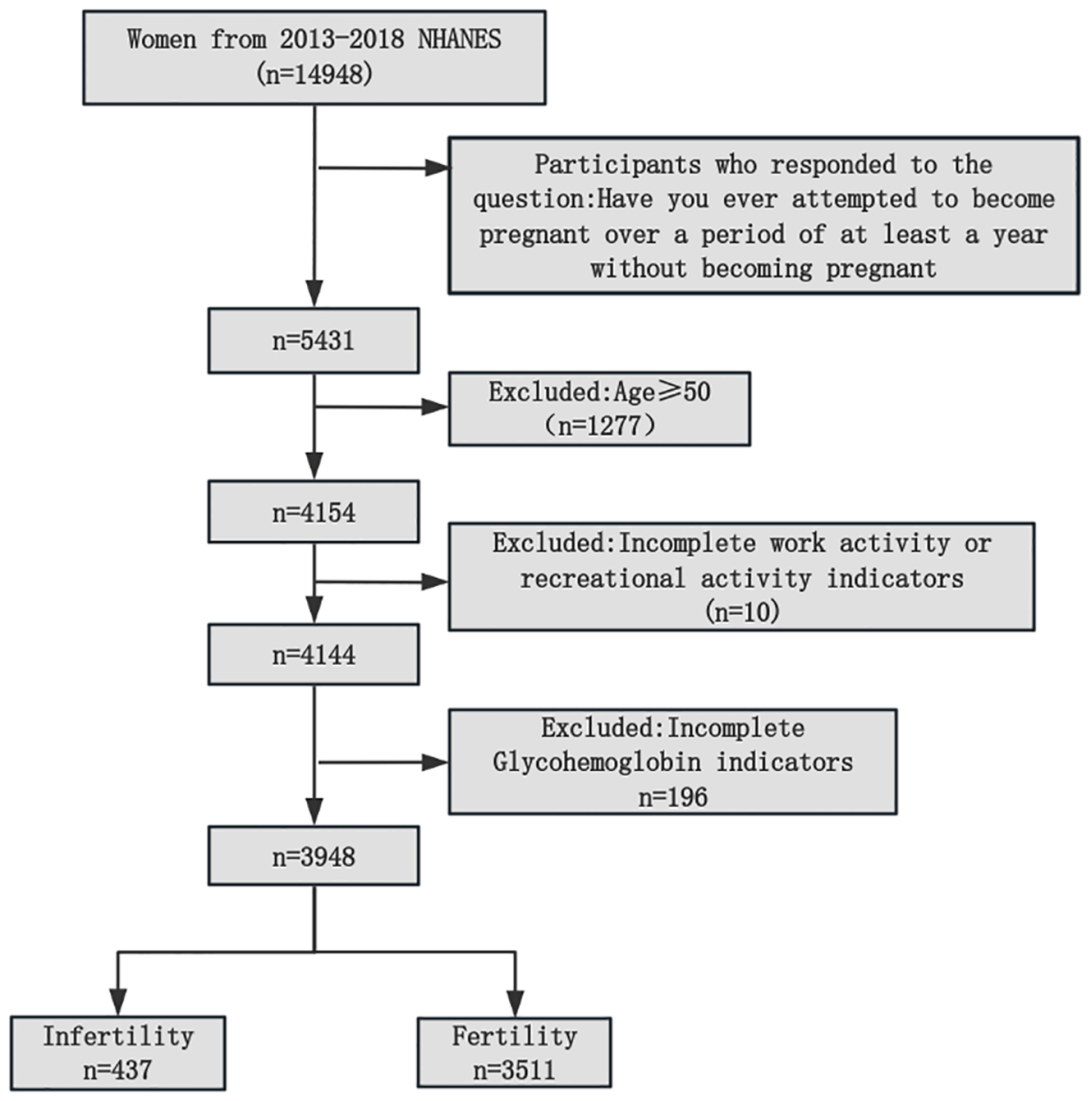
Flowchart of participants selection from the NHANES 2013–2018. NHANES, National Health and Nutrition Examination Survey.

This study utilized cross-sectional survey data from the NHANES (https://wwwn.cdc.gov/nchs/nhanes/). A total of 14,948 women were included from 3 cycles of the NHANES, and 5,431 of them responded to the question on infertility. After excluding individuals aged 50 years or older who did not have exercise patterns or HbA1c indicators, the study included a total of 3,948 participants, as visually demonstrated in **Figure 1**. The NHANES protocols were approved by the NCHS Research Ethics Review Board, and written informed consent forms were provided by all of the participants.

### Assessments of PA, infertility and HbA1c

The NHANES study collected data on PA via a questionnaire, in which individuals reported their own PA levels. The questions regarding PA were as follows: “Do you participate in any vigorous exercise or fitness activity on a regular basis that leads to a significant increase in your breathing or heart rate, such as running or playing basketball for at least 10 minutes?”; and “Do you often participate in any type of moderate-intensity PA, such as brisk walking, cycling, swimming, or playing volleyball, for at least 10 minutes, which causes a slight increase in your breathing and heart rate?”. This study included both work and recreational activities. According to the recommendations of WHO and national health institutions, adults aged 18–64 years need to engage in at least 150 minutes of moderate-intensity aerobic exercise (such as brisk walking or cycling) or 75 minutes of high-intensity aerobic exercise (such as running or swimming) per week to promote their health. We separately calculated the durations of both moderate- and high-intensity PA and classified PA into three categories: inactive, less active (≤ recommended amount of activity), and active (≥ recommended amount of activity) (**Table 1**). We reported infertility via the following question: “Have you made any attempts to conceive for a duration of at least one year without achieving pregnancy?”. We classified those individuals who responded affirmatively as infertile and those who responded negatively as fertile. We classified unanswered or refused responses as missing values. At Columbia University, Missouri, HbA1c tests were conducted. The A1c G7 HPLC Glycohemoglobin Analyzer (produced by Tosoh Medicine, Inc., 347 Oyster Pt. Blvd., Suite 201, So. San Francisco, CA 94080) was used to measure HbA1c levels.

**Table 1:** Physical activity (PA) classification.

INACTIVE PA	LESS ACTIVE PA	ACTIVE PA
No high-intensity PA	Only high-intensity PA, and the weekly PA time is less than 75 minutes.	Only high-intensity PA, and the weekly PA time must exceed 75 minutes.
No moderate-intensity PA	Only moderate-intensity PA, and the weekly PA time is less than 150 minutes.	Only moderate-intensity PA, and the weekly PA time must exceed 150 minutes.
Sedentary behavior	The duration of both types of PA is less than 75 minutes for high-intensity PA and less than 150 minutes for moderate-intensity PA.	The weekly duration of high-intensity PA exceeds 75 minutes, and the moderate-intensity PA also exceeds 150 minutes minutes.

### Other variables of interest

We conducted a thorough examination of 10 potential variables that could influence the relationship between physical activity PA and infertility. These variables included age (18–49 years), race, sex, BMI, smoking status, education, sleep, history of diabetes, poverty-to-income ratio (PIR), menstrual cycle, current pregnancy, and HbA1c. To facilitate comparative analysis, we converted continuous variables into categorical variables and divided each covariate into a reference group and additional groups for comparison. Furthermore, we designated “unknown, rejected, or undetectable” values as values that were not present or could not be determined.

### Statistical analysis

The chi-square test was used to assess the statistical disparities in confounding factors between the two groups, including fertility and infertility. A logistic regression model was used to investigate the correlation between infertility and PA. The model calculated the odds ratio (OR) between infertility and various categories of work and recreational PA, and it compared the 95% confidence interval (CI) with that of women who were inactive (the reference group). The regression model was adjusted for age, race, BMI, smoking status, education, sleep, history of diabetes, poverty-to-income ratio (PIR), menstrual cycle and current pregnancy or nonpregnancy status. In Model 1, no variables were adjusted; in Model 2, racial and educational adjustments were made; and in Model III, we accounted for all of the covariates and subsequently examined them as subgroup variables in a stratified analysis. To assess the extent to which HbA1c mediates the association between PA patterns and infertility, we performed a causal mediation analysis. Initially, we employed the causal stepwise regression test to conduct linear regression and logistic regression on the dependent variable with respect to the independent variable (17). We subsequently determined the regression coefficients a, b, c, and c′ in the mediation model. The RMediation package in R software was used to calculate the 95% confidence interval of Za * Zb via the cumulative distribution approach (18). The PA pattern, infertility status, and HbA1c level were used to represent the independent variables, outcome variables, and mediating variables, respectively, in our survey. A significance threshold of P<0.05 and a Za * Zb 95% CI excluding 0 guided the determination of statistical significance.

## Results

### Characteristics of participants

This study included 3,948 female participants, with an average age of 33.24 ± 9.47 years; additionally, 11.07% had previously experienced infertility. **Table 2** presents a thorough summary of the fundamental characteristics of the participants. Women who were over the age of 35 years, of non-Hispanic white ethnicity, and obese, in addition to having a high poverty-to-income ratio (PIR) and a history of diabetes, were more likely to experience infertility. There was no statistically significant disparity between the two groups in terms of education level, smoking status, sleep duration, or regularity of the menstrual cycle. Furthermore, individuals with infertility exhibited a notable increase in HbA1c levels compared to patients who did not have a history of infertility (2.47 ± 0.17 vs. 2.43 ± 0.2).

**Table 2:** Baseline characteristics of study population according to fertity*.

	Infertility (n=437)		Fertility (n=3511)		P Value†
	Mean/n	SE/%	Mean/n	SE/%	
Age (n %)					<0.001
<35	162	7.7	1941	92.3	
≥35	275	14.9	1570	85.1	
Race (n %)					0.02
Mexican-American	62	9.0	629	91.0	
Other Hispanic	38	9.2	375	90.8	
Non-Hispanic White	174	13.2	1141	86.8	
Non-Hispanic Black	90	10.6	759	89.4	
Other Race	73	10.7	607	89.3	
BMI (n %)					<0.001
Normal<25	119	8.7	1256	91.3	
Overweight(25-29.99)	77	8.3	852	91.7	
Obesity(≥30)	234	14.6	1373	85.4	
Education (n %)					0.12
<Highschool	59	10.1	524	89.9	
≥Highschool	371	12.4	2622	87.6	
Smoking (n %)					0.65
Every day	76	14.0	467	86.0	
Some day	16	12.0	117	88.0	
Not at all	66	15.2	369	84.8	
Sleeping (n %)					0.45
≤8	122	10.6	1028	89.4	
>8	131	9.7	1221	90.3	
Diabete history (n %)					<0.001
Yes	39	19.8	158	80.2	
No	388	10.5	3298	89.5	
PIR (n %)					0.04
<1.3	134	10.1	1191	89.9	
1.30-2.99	117	10.7	977	89.3	
≥3.0	159	13.2	1045	86.8	
Menstrual cycle (n %)					0.54
Regular menstruation	385	11.2	3056	88.8	
Irregular menstruation	52	10.3	454	89.7	
Is currently in a pregnant state? (n %)					0.72
YES	18	14.2	109	85.8	
NO	243	13.0	1620	87.0	
HbA1C (mmol/L)	2.47	0.2	2.43	0.17	<0.001

BMI, body mass index; PIR, poverty income ratio; HbA1C, glycosylated hemoglobin.

*Values presented are means and their SE for continuous variables, and as percentages for categorical variables.

†We tested for differences in baseline characteristics using weighted χ 2 tests for categorical variables and weighted ANOVA for continuous variables.

### Results of multiple logistic analysis

These findings indicated that there is no substantial association between work-related PA and infertility. However, in recreational PA, there is no substantial correlation between less active PA and infertility, whereas there is a strong association between active PA and infertility. During active PA, the likelihood of infertility is 0.79 times that during inactive PA. In Model II, after accounting for the confounding factors of race and education, the correlation remained significant (OR=0.75, 95% CI: 0.59, 0.96; *p*=0.02). However, in Model III, after adjusting for confounding factors in all of the covariates, the strength of this correlation was no longer statistically significant (OR = 0.82, 95% CI: 0.38, 1.78; *p* = 0.62), as indicated in **Table 3**.

**Table 3:** Association between PA patterns and infertility.

	Model I	Model II	Model III
	OR 95%CI P Value	OR 95%CI P Value	OR 95%CI P Value
Work related PA			
Inactive	Ref	Ref	Ref
Less active	0.87 (0.65-1.16) 0.33	0.80 (0.60-1.08) 0.15	0.79 (0.33-1.86) 0.59
Active	1.01 (0.80-1.28) 0.92	0.94 (0.74-1.20) 0.63	0.91 (0.46-1.79) 0.78
Recreational PA			
Inactive	Ref	Ref	Ref
Less active	0.89 (0.69-1.14) 0.36	0.84 (0.65-1.09) 0.20	0.66 (0.28-1.60) 0.36
Active	0.79 (0.62-1.0) 0.045	0.75 (0.59-0.96) 0.02	0.82 (0.38-1.78) 0.62

PA, physical activity.

Model 1 adjusted for none.

Model 2 adjusted for race and education.

Model 3 adjusted for age, race, BMI, education level, sleeping hour, smoke status, Diabete history, PIR, menstrual cycle, pregnant state.

### Stratified analyses

A stratified analysis of the correlation between all of the covariates and infertility in three different modes of recreation-related phsical activity (PA) revealed that among women who engaged in active and less active PA, obese women (BMI ≥ 30) had a greater probability of infertility than women of normal weight; however, this difference was not observed in women without exercise. In addition, there was no statistically significant difference in the incidence of infertility between overweight women (BMI = 25–29.9) and normal-weight women among the three PA modes. More significantly, there was a significant correlation between a history of diabetes and infertility in women without exercise and in women with active PA; however, this correlation was not significant in women with less active PA, as indicated in **Table 4**.

**Table 4:** Recreational PA patterns – infertility subgroup analysis.

	Inactive (n=1823)	Less active (n=893)	Active (n=1232)
	OR 95%CI P Value	OR 95%CI P Value	OR 95%CI P Value
Age (Y)			
<35	Ref	Ref	Ref
≥35	1.97 (1.47-2.65) <0.001	2.12 (1.37-3.27) <0.001	2.21 (1.51-3.24) <0.001
Race			
Mexican-American	Ref	Ref	Ref
Other Hispanic	1.19 (0.68-2.07) 0.54	1.27 (0.50-3.27) 0.62	0.60 (0.23-1.56) 0.29
Non-Hispanic White	1.61 (1.06-2.44) 0.03	1.72 (0.84-3.56) 0.14	1.50 (0.84-2.70) 0.17
Non-Hispanic Black	1.03 (0.65-1.63) 0.92	1.61 (0.73-3.58) 0.24	1.34 (0.70-2.60) 0.38
Other Race	1.38 (0.85-2.24) 0.19	1.11 (0.49-2.50) 0.81	1.17 (0.66-1.17) 0.66
BMI			
Normal<25	Ref	Ref	Ref
Overweight (25-29.99)	0.87 (0.57-1.33) 0.51	1.35 (0.71-2.57) 0.36	0.80 (0.45-1.42) 0.45
Obesity (≥30)	1.29 (0.93-1.80) 0.14	2.51 (1.50-4.19) <0.001	2.30 (1.50-3.53) <0.001
Education			
<Highschool	Ref	Ref	Ref
≥Highschool	1.32 (0.93-1.88) 0.13	1.04 (0.50-2.16) 0.91	1.76 (0.80-3.88) 0.16
Smoking			
Every day	Ref	Ref	Ref
Some day	0.97 (0.43-2.18) 0.94	0.83 (0.25-2.77) 0.76	0.78 (0.24-2.54) 0.68
Not at all	0.83 (0.49-1.40) 0.48	1.40 (0.64-3.07) 0.40	1.49 (0.72-3.07) 0.28
Sleeping (H)			
<8	Ref	Ref	Ref
≥8	1.06 (0.74-1.54) 0.74	0.77 (0.44-1.34) 0.35	0.76 (0.46-1.26) 0.29
Diabete history			
NO	Ref	Ref	Ref
YES	1.98 (1.21-3.24) 0.006	1.43 (0.62-3.27) 0.40	3.22 (1.54-6.75) 0.002
PIR			
<1.3	Ref	Ref	Ref
1.30-2.99	1.11 (0.80-1.58) 0.56	0.79 (0.43-1.45) 0.45	1.29 (0.77-2.19) 0.34
≥3.0	1.69 (1.12-2.41) 0.004	1.25 (0.74-2.09) 0.40	1.28 (0.78-2.09) 0.32
Menstrual cycle			
Irregular menstruation	Ref	Ref	Ref
Regular menstruation	1.11 (0.75-1.65) 0.59	1.30 (0.65-2.57) 0.46	1.69 (0.84-3.41) 0.14
Is currently in a pregnant state?			
NO	Ref	Ref	Ref
YES	1.04 (0.50-2.14) 0.93	1.63 (0.64-4.12) 0.31	0.71 (0.23-2.70) 0.71

### Mediation analysis

**Figure 2:**
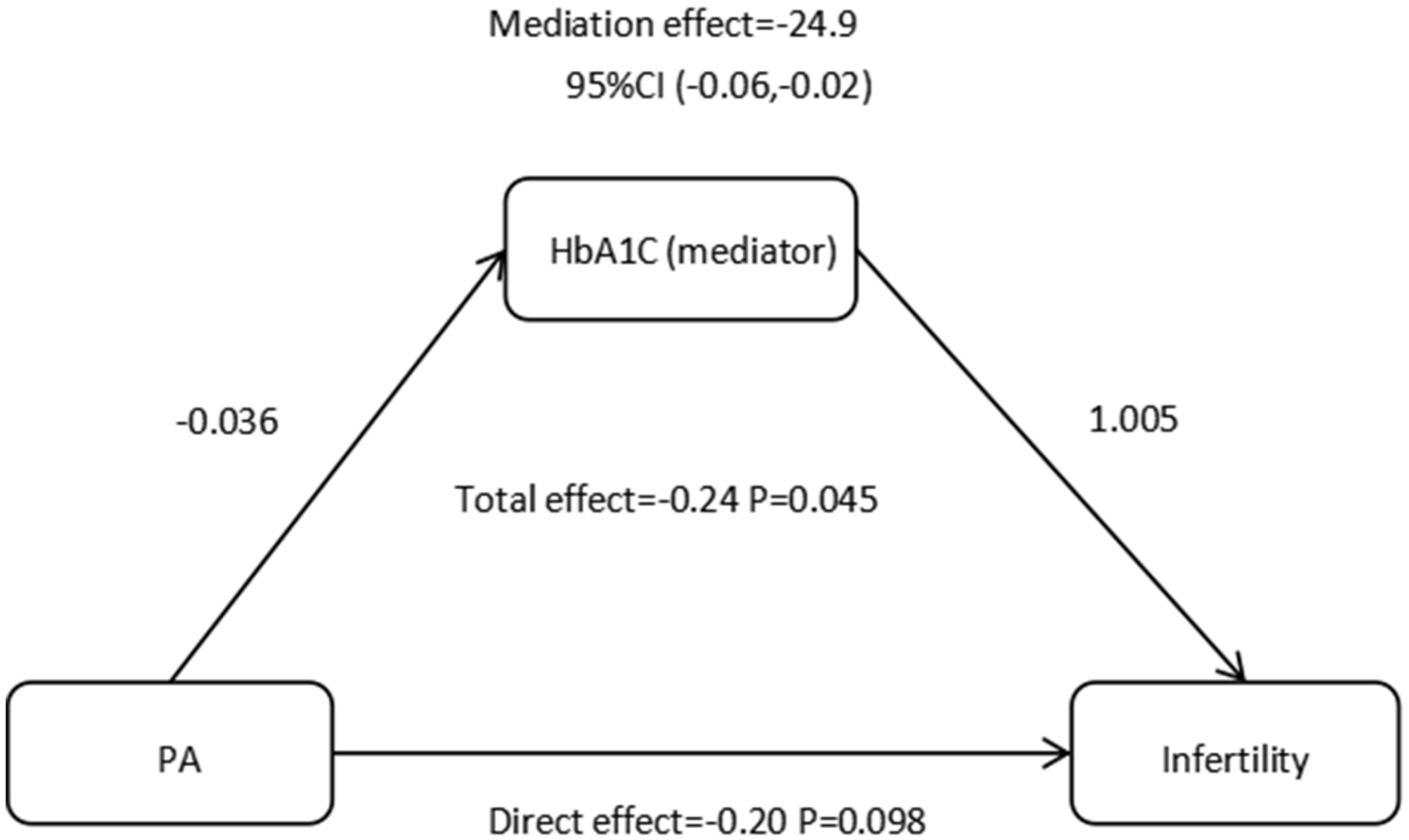
Effect of the HbA1C (mediators) on the relationship between PA (exposure) and infertility (outcome). HbA1C, glycosylated hemoglobinl; PA, physical activity.

Mediation analysis sought to measure the extent to which HbA1c acts as a mediator in the relationship between recreational physical PA patterns and infertility. In contrast to conventional mediation research, our study specifically examined categorical variables as both independent and dependent factors. Thus, we calculated the relative mediation effects, relative direct effects, and relative total effects and computed the mediating effect value by multiplying Za and Zb, after which we assessed the 95% confidence interval of Za * Zb via the product distribution method. The results of our study indicated that engaging in recreational active PA does not have a statistically significant direct effect on infertility (*p*=0.098). However, we found that HbA1c plays a mediating role in the relationship between recreational active PA and infertility, with a mediating effect of -24.9 (95% CI: -0.06, -0.02), as shown in **Figure 2**.

## Discussion

As society progresses and educational levels improve, an increasing number of individuals over the age of 35 express their desire to become pregnant, and infertility is particularly common among older women. While aging is the primary cause of declining fertility, the development of assisted reproductive technology in recent years has provided the possibility of reproduction for older infertile women. As a result, physiological and individual factors play an important role for women of advanced reproductive age. Our study extended the age range of the research subjects to females aged 18 to 49, potentially increasing the universality and application of the research findings, allowing them to span a broader range of female populations and providing more thorough data support for clinical treatment. This study primarily investigated the role of HbA1c in modulating the link between PA and infertility. We performed a cross-sectional study utilizing a questionnaire survey to examine the interrelations among PA, HbA1c, and infertility within a population that willingly participated in the survey in the United States throughout the same period of time. The incidence of infertility is lower with active PA, as recommended by WHO, than with inactive PA. However, this tendency was solely evident in recreation-related PA, with no differences in infertility rates being associated with work-related PA. Moreover, HbA1c is negatively correlated with PA and positively correlated with infertility, and it may mediate the link between PA and infertility. The impact of obesity and a history of diabetes on infertility is primarily mediated by PA.

By distinguishing our study from previous studies, we specifically offer a novel perspective by categorizing PA patterns in accordance with World Health Organization(WHO) guidelines. This choice was based on the clear notion that neither high-intensity nor moderate PA encompasses the duration of the activity. Research has indicated that moderate PA enhances pregnancy rates in women with reproductive problems and that consistent moderate PA supports reproductive health maintenance (19–21). HaKimi et al. analyzed an extensive amount of literature and proposed that exceeding 60 minutes of strenuous exercise on a daily basis increases the chance of anovulation; however, 30–60 minutes of moderate-intensity exercise may mitigate ovulatory infertility. Therefore, following the WHO guidelines, we classify exercise into inactive, less active, and active exercise types while considering both exercise intensity and duration (22).

Our research indicated that the relationship between different types of physical PA and infertility is inconsistent. recreation-related PA is strongly associated with reduced infertility rates, which is consistent with the findings of previous studies that highlighted its importance for improving reproductive health in women of childbearing age (23). We propose that this effect is due to the advantageous benefits of consistent recreational activity, as recommended by WHO. This may mitigate the adverse effects of an energy deficit on fertility by increasing the body’s metabolic rate, regulating endocrine function and strengthening immunity (24). However, such nonlinear associations have not been recognized between work-related PA and infertility. Research on the relationship between work-related PA and infertility has produced inconsistent findings. Some evidence indicated that increased levels of work-related PA are associated with a greater risk of infertility (25). However, other studies suggested that insufficient work-related PA may adversely affect fertility (26). Furthermore, our research revealed no substantial correlation between work-related PA and infertility. We have analyzed potential explanations and shown that PA predominantly affects female reproductive health via metabolic control, whereas work activities denote obligations that individuals must fulfill during working hours, which often entails extended and low-intensity static tasks. Unlike leisure activities, which are predominantly subjective, work-related PA may not aid in metabolic regulation. A study of Taiwanese workers also revealed no significant relationship between work-related PA and metabolic markers (27, 28). This may also be linked to endocrine regulation. Research has indicated that PA augments the body’s biosynthetic response by increasing the levels of growth hormone and insulin-like growth factor, thus promoting reproductive health (29). The research by Schjeldrup Skarpsno (30) suggested a correlation between professional activity and insomnia, which clearly affects endocrine function.

Previous studies have reported that abnormal HbA1c is related to pregnancy problems. High HbA1c can increase the risk of gestational diabetes mellitus (31), and may also increase the risk of early abortion and premature birth (32, 33). Most importantly, our research suggests that HbA1c may play a role in the connection between recreation-related PA and infertility. Our data indicate that PA can downregulate HbA1c and has a negative correlation with infertility, which is consistent with the findings of previous studies (34, 35). HbA1c serves as a diagnostic tool for monitoring diabetes. Numerous studies have investigated the effects of a physically active lifestyle on individuals who are diagnosed with diabetes (36, 37). A previous study revealed that children diagnosed with type 1 diabetes who had lower levels of PA had poorer blood glucose control than did children who participated in more consistent PA. The level of HbA1c was significantly elevated in the less physically active group compared with the more physically active group (38). An analysis of a population sample of women aged 18–45 years in the United States revealed a notably positive association between HbA1c levels and infertility (39). However, this study investigated the role of HbA1c in influencing the association between PA patterns and infertility. We identified the mediating regulatory function of HbA1c. The findings of this study demonstrated that recreation-related PA does not have a statistically significant direct effect on infertility, as evidenced by a coefficient of -0.20. These findings suggest that the influence of exercise on infertility is consistently moderated by additional factors. The statistical analysis demonstrated that the mediating effect of HbA1c was significant, as evidenced by a coefficient of -24.9. These findings provide some evidence for the potential role of HbA1c in the modulation of PA on infertility, but more research is required to establish a definitive conclusion.

Finally, we unexpectedly found that the influence of obesity and a history of diabetes on infertility is mainly regulated by PA. The impact of obesity on the incidence of infertility mainly occurs in the active and less active population, thus indicating that weight loss in women who exercise is beneficial for their reproductive health, whereas weight loss alone does not promote female reproductive health in women who do not exercise. This may indicate that simply losing weight is not related to promoting reproductive health. A large amount of clinical data has confirmed that weight loss can improve reproductive function, and weight loss has become a basic component of infertility treatment for obese individuals (40). However, previous research has shown that increasing weight loss in obese or overweight women before IVF treatment does not improve reproductive outcomes (41). Our research results also confirmed this finding. In addition, among the less active population, a history of diabetes does not affect the incidence of infertility, which indicates that less activity may be beneficial to women with a history of diabetes.

Our research provides further evidence for the negative correlation between PA and infertility; moreover, based on previous reports, we found no significant correlation between work-related PA and infertility incidence. These findings suggest that recreation-related PA has greater significance in promoting reproductive health. In addition, mediation analysis revealed potential reasons for the association between PA and infertility. Based on these data, it will be possible to provide patients with more accurate and evidence-based advice to determine the optimal frequency and intensity of exercise, thus increasing their chances of pregnancy. Finally, the findings of hierarchical analysis also suggest that, in our clinical research, the body mass index of individuals who exercise is more worthy of attention, and less active PA is more recommended for women with a history of diabetes.

However, this study has several significant drawbacks. The cross-sectional approach that was used in our study did not allow us to establish a cause-effect association between PA and infertility. To confirm this relationship, additional prospective cohort studies or randomized controlled trials are needed. Furthermore, the identification of diseases depends on self-reported responses from participants, which could lead to the introduction of recall bias and misclassification. Nevertheless, prior research has demonstrated that data obtained from the NHANES are reliable for evaluating the prevalence of infertility in the entire population (42, 43). Finally, future research might look into the reasons and treatment methods of infertility in women of different ages, as well as how to provide more individualized fertility support to women of all ages.

In conclusion, the findings of this study revealed that confounding factors had a significant influence on the correlation between the two variables. Although stratified analysis has been conducted, further inquiries are needed to explore the relationships between confounding factors and infertility. Although mediation analysis is essential, it is worth noting that the results obtained within the cross-sectional framework are still correlated and do not prove causal relationships.

## Conclusion

Our results revealed that HbA1c may play a potencial mediation role in the association between PA and infertility, thus revealing a potential mechanism underlying the association between PA and infertility, which offers valuable insights for establishing healthy PA guidelines for women. However, due to the fact that this study was a cross-sectional study, more prospective cohort studies are needed in the future to explore causality.

## Data Availability

The original contributions presented in the study are included in the article/**Supplementary Material**. Further inquiries can be directed to the corresponding author/s.
