# Supplementary material for: The association between glycated hemoglobin, physical activity and infertility: a multiple logistic regression and mediation analysis based on the NHANES database
Source: Front Endocrinol (Lausanne). 2025 Mar 26;16:1495470. doi: 10.3389/fendo.2025.1495470 (PMC11980949; doi:10.3389/fendo.2025.1495470)
Supplement: Supplementary file 1 [file DataSheet1.pdf]

causal stepwise regression test

Model 1：Y=cX+e1; X is independent variable ; Y is dependent variable; c is total effect
We used logistic regression to calculate the value of c
c=-0.240 P=0.045

Model 2: M=aX+e3 ; M is Mediating variable; X is independent variable;
We used linear regression to calculate the value of a
a=0.036 P<0.05

Model 3 ：Y=C'X+bM+e2; Y is dependent variable; X is independent variable; M is Mediating variable; C' is dirrect effect;
We used logistic regression to calculate the value of C' and b
C'=-0.20 P=0.098; b=1.005 P<0.05

Za=a/SE(a)=-0.036/0.006=-6
Zb=b/SE(b)=1.005/0.242=4.15
Za.Zb=-24.9

cumulative distribution approach

1. install.packages("RMediation")

2. library(package="RMediation")

3. medci(mu.x=-0.036,mu.y=1.005,se.x=0.006,se.y=0.242,rho=0,alpha=0.1,type="prodclin") $`90% CI`

[1] -0.05898035 -0.01719332

$Estimate

[1] -0.03618

$SE

[1] 0.0106943
